# ‘Space to hide’: experiences of remote provision across child and adolescent mental health services (CAMHS)

**DOI:** 10.1186/s12913-022-08806-5

**Published:** 2022-11-14

**Authors:** Joanne Worsley, Shaima Hassan, Lisa Nolan, Rhiannon Corcoran

**Affiliations:** 1 Department of Primary Care and Mental Health, University of Liverpool, Liverpool, UK; 2 NIHR Applied Research Collaboration North West Coast (ARC NWC), Liverpool, UK; 3 NHS Cheshire and Merseyside Integrated Care Board, Liverpool Place, UK

**Keywords:** Mental health, CAMHS, Remote provision, Accessibility, Flexibility, Therapeutic relationships, Non-verbal communication

## Abstract

**Background:**

The global COVID-19 pandemic necessitated rapid adoption of remote provision across child and adolescent mental health services (CAMHS). The study aimed to understand young people’s, parents’/carers’, and professionals’ experiences of remote provision across CAMHS in one NHS Trust in the North West of England to inform future recovery practice so that remote sessions can continue where they have been well received but re-thought or replaced where they have not.

**Methods:**

The study sample comprised three groups: (i) young people, (ii) parents/carers, and (iii) clinical staff. Semi-structured interviews and focus groups were used to collect data. Data were analysed using thematic analysis.

**Results:**

Three overarching themes were identified: ‘Remote therapeutic experiences’; ‘Spaces and places of therapy’; and ‘Future of CAMHS’. Although remote appointments increased flexibility within the service, the quality of the relational experience was altered, typically for the worse. Clinicians felt less able to examine vital forms of non-verbal communication, which were considered instrumental in assessing and engaging people experiencing difficulties, leaving some questioning their professionalism. Although some young people suggested that remote provision increased comfort levels, others felt their place of comfort and safety was invaded.

**Conclusions:**

Reduced travel time for both clinicians and families may increase capacity, enabling the service to meet the increased demand if clinical effectiveness can be preserved. In considering future models of provision, assessing clinical need, patient and family preference, and access to space and hardware are all critical when deciding which modality to use for the best outcomes for each individual.

## Introduction

The COVID-19 pandemic has been an unprecedented challenge for healthcare provision. According to a survey conducted by the World Health Organisation (WHO), the COVID-19 pandemic disrupted or halted mental health services in 93% of countries worldwide [1]. In response to this global pandemic, the UK government issued a nationwide lockdown to suppress virus transmission. Children and young people (CYP) in particular have been adversely affected by the control measures, through closure of schools and separation from friends [2, 3]. Although children and young people’s mental health was deteriorating before the COVID-19 crisis [4–7], the ongoing impact of the pandemic on young people’s mental health and wellbeing is a cause for concern (see [8]).

Child and Adolescent Mental Health Services (CAMHS) are NHS-funded statutory services in the UK, which assess and support children and young people with mental health difficulties [9]. Although CAMHS services have traditionally been delivered face-to-face, a number of online youth counselling services operated before the global COVID-19 pandemic, such as kooth.com (a youth counselling service providing online counselling and support for young people aged 11–25 years in certain regions of the UK). As previous research suggests that practitioners spend more time building rapport online than accomplishing tasks [10], the therapeutic process may differ in this environment. Nevertheless, research suggests that therapeutic alliances sufficient to facilitate psychological change appear possible online [11].

The global COVID-19 pandemic necessitated the rapid adoption of remote provision across CAMHS [3, 12, 13]. To explore the impact of the COVID-19 pandemic on service provision and how this affected patient, family, and staff experiences, a service evaluation of a CAMHS eating disorder service in the North of England was conducted [14]. This evaluation indicates that although face-to-face was the preferred type of consultation, service satisfaction did not change during the COVID-19 lockdown period. The flexibility to use different mediums improved access; however, the quality of the relational experience was altered online [14]. According to one study exploring practitioners’ experiences of remote consultations across one East of England CAMHS, the pivot to remote provision did not adversely affect rapport, safeguarding, or risk assessment [12]. Conversely, Bentham and colleagues found that clinicians lacked the training and guidance to maximise the effectiveness in completing their role [15]. As telephone calls were the predominant format of appointments, clinicians felt less able to examine vital forms of non-verbal communication [15] which were considered instrumental in assessing and engaging people experiencing difficulties. Healthcare professionals often rely on forms of non-verbal communication to form clinical impressions and maintain engagement [15]. As research in this area has predominantly focused on the perspectives of practitioners and clinicians, it is important to explore young people’s and parents/carers’ perspectives of remote provision, especially as future delivery models should be designed and commissioned with young people’s voices in mind.

Through qualitative means, the present study aimed to understand young people’s, parents/carers’, and professionals’ experiences of remote provision across CAMHS in one NHS trust in the North West of England. This approach was selected as appropriate to provide a level of rich lived experience detail from the perspectives of those accessing, or delivering, remote CAMHS provision that was sufficient to inform the nature of future delivery, to highlight strengths to promote and pitfalls to avoid. In the Trust in question, community multi-disciplinary CYP mental health teams work with children and young people with varying issues, such as low mood, anxiety, self-harm, suicidal ideation, behavioural difficulties, and trauma. The specialist teams provide access to assessment and intervention, and most commonly offer cognitive behavioural therapy (CBT), family and systemic therapy, trauma focused therapies, psychodynamic psychotherapy, interpersonal therapy, art therapy, psychiatry, and medication management. The Trust in question set up a 24/7 crisis care line in the face of the pandemic for CYP experiencing mental health crisis. It is important to understand the clinical settings and patient groups for whom remote provision is most appropriate and for whom remote provision does not work, as this knowledge will inform subsequent service delivery. The purpose of this study therefore is to inform future recovery practice so that remote sessions can continue where they have been well received but re-thought or replaced where they have not.

## Methods

### Ethical approval

Ethical approval was received from Preston Research Ethics Committee (IRAS ID: 297792). All methods were carried out in accordance with relevant guidelines and regulations, and reporting guidelines for qualitative research were followed. All participants involved in the research provided written informed consent. Informed consent from a parent or legal guardian was also obtained when required.

### Participants

Posters advertising the study were circulated by CAMHS professionals. This recruitment approach was used across all stakeholder groups and was selected as a means of summarising the research project succinctly in a visual way in order to engage potential participants. All individuals who responded to the poster were invited to participate in the study. A total of 22 participants took part: 10 young people (see Table 1 for CYP characteristics), 9 healthcare professionals (5 females and 4 males), and 3 female parents/carers. All participants had experience of CAMHS prior to, and during, the COVID-19 pandemic. Young people were remunerated for their time.

**Table 1:** CYP characteristics (gender and age)

CYP participant number	Gender	Age
CYP 01	Female	16
CYP 02	Female	16
CYP 03	Gender neutral	15
CYP 04	Female	20
CYP 05	Male	15
CYP 06	Female	15
CYP 07	Female	18
CYP 08	Female	16
CYP 09	Male	16
CYP 10	Female	Undisclosed

### Data collection

Semi-structured telephone or video call interviews and focus groups were conducted using a prepared topic guide that had been co-produced with children and young people, parents/carers, a mental health promotion worker, and a CAMHS assistant psychologist. The guide comprised questions about the impact of COVID-19 on usual provision; the alternative measures put in place to try to continue service provision; and individual’s experiences of using or delivering these services. CYP answered questions independently of parents’ input and were given the option of participating in a focus group (alongside other CYP) or a one-to-one interview. Of those who participated, five CYP opted to take part in a focus group and five CYP opted to take part in a one-to-one interview. All healthcare professionals and parents/carers took part in a one-to-one interview. All interviews and focus groups were conducted by the first author who had no prior relationships with the participants. The first and second author co-facilitated the focus group with young people to ensure that there was an appropriate member of staff available if any participant experienced distress during the group discussion. During the focus group, the researchers acted as ‘facilitators’, encouraging interactive discussion while keeping their own inputs light touch. Data collection was carried out during October 2021 to March 2022. All interviews lasted approximately 30 min while the focus group lasted approximately one hour. With participant consent, the focus group and interviews were recorded and transcribed verbatim. All transcription was undertaken by the first author to allow maximum data immersion.

### Analysis

Thematic analysis, outlined by Braun and Clarke [16], is a qualitative method that aims to identify and report recurrent themes in data. The first author kept a reflexive diary throughout the research process. Taking notes throughout the data collection period was important as a means of capturing observations about participants’ body language and facial expressions. Contextual and reflexive notes were subsequently documented in interview transcripts to facilitate data analysis. Initial and repeated reading of the transcripts was undertaken by the first author, considering both contextual and reflective notes. Although there were some pre-determined areas the researcher wanted to explore, a largely inductive approach was used to reflect on unexpected concepts within the data. Line-by-lining coding was undertaken by the first author. A subset of transcripts were read and coded independently by the second author. The codes were agreed on by the first and second author (both trained and experienced in qualitative data analysis), and grouped together into categories, which were subsequently grouped into themes that triangulated across stakeholder groups. Themes and subthemes were renamed, refined, and agreed by the whole research team, ensuring that the final analysis did not solely reflect the personal interpretation of one team member.

## Results

Analyses of the focus group and interview data across the stakeholders revealed three overarching themes summarised in Table 2.

**Table 2:** Overarching themes and subthemes

Themes	Subthemes
Remote therapeutic experiences	Accessibility and flexibility
	Translating the elements of therapy
	Practical issues
	Losing the cues
	Drivers of trust and rapport
Spaces and places of therapy	Spaces of comfort and safety
	‘When you’re at home it’s home time’
	‘You lack privacy to speak about things’
Future of CAMHS	Moving to a hybrid model of provision
	Efficacy
	Increased demand: Is social prescribing a solution?

## Theme 1: Remote therapeutic experiences

### Accessibility and flexibility

In the face of COVID-19, CAMHS have innovated rapidly, notably with accelerated digitalisation of services in their array of forms and formats (e.g., ‘The NHS had their own platform developed called Attend Anywhere’ (HCP 06) and ‘We set up a 24-h crisis service for CAMHS, a telephone crisis service in a very short space of time’ (HCP 04)). New ways of working have been introduced in the face of the COVID-19 pandemic, which have improved ease of access:

It's helped some families have an easier access to CAMHS. So, either through the crisis line, or actually not having to get three buses across town to come to an appointment (HCP 04).

As young people were no longer required to travel to CAMHS buildings, appointments were not constrained by room availability on site. Staff therefore had more flexibility to offer appointments:

There's some increased capacity from doing it online, because in the building, we're quite limited in terms of the number of rooms available so you can be more flexible about when you can offer them appointments online (HCP 01).

Young people and parents/carers found online provision more ‘convenient’ (HCP 01) in comparison to attending in-person appointments, especially as remote provision reduced travel time and the associated costs:

They don’t have to come all the way to [name of NHS Trust], which probably takes a couple of hours by the time you've got out of school, driven there, parked, walked all the way across to the [name of CAMHS] building. Being able to do it from home without having to interrupt their day (HCP 01).

The biggest thing is no commute… For children who live far away from services having to get the train and having to pay a train fare, or put petrol in the car, it's decreasing accessibility to the service (CYP 04).

As engaging online mitigates practical and psychological barriers to full engagement with services, these new ways of working may be beneficial for certain individuals in particular, such as those with agoraphobia or practical issues that limit their ability to travel:

[It] got to the point where she didn’t want to leave the house… Her anxiety hit the roof… I wouldn’t have been able to get her to a face-to-face meeting… If there are kids out there with that anxiety where they can’t leave the house… I think this is the answer for them (Parent 01).

Remote provision has also made it possible for young people to attend therapeutic intervention sessions from school or college, with little disruption to their usual routine as ‘they are not then losing much of their day and the important routine and structure’ (HCP 02):

We are doing video calls at the schools a lot more. I suppose it’s flexible, that’s how the service has changed (HCP 05).

It's a lot easier to do it over the phone or over Teams than it is to actually go into CAMHS… It’s flexible… I can do a CAMHS call in college (CYP 02).

Last, for healthcare professionals, remote working has made meetings, both internal and external, easier through reducing travel time. Staff reported that virtual meetings meant that they could join from anywhere, and many found these meetings more efficient and less time consuming. For example, ‘systemic stuff, it's so much easier and it saves so much time’ (HCP 07). This new way of meeting also facilitated easy access for professionals from other trusts:

I think that's loads better because we're now having [multidisciplinary team] meetings easily, involving lots of people across Trusts (HCP 02).

Another positive is meetings such as social care meetings or school meetings, it’s easier as you haven’t got to travel to a school… Generally, online they are a bit quicker as well because there is no small talk (HCP 05).

### Translating the elements of therapy

In the face of COVID-19, therapies originally designed to be delivered face-to-face were translated into online formats. However, clinicians have struggled to recreate certain elements:

For those of us who are in the creative therapies space, it is much more tricky because our medium is that we rely on proximity to people to pick up nonverbal communication using action methods. Even if you think about role playing in a group, it's very tricky to do that online (HCP 04).

It feels like it's harder to be creative. It's harder to draw on all the things that you might be able to do face-to-face. So, I normally work doing a lot of things visually, writing things down, drawing things out, physically working on something together… Being able to do that two way was just lost (HCP 09).

She was giving me DBT [Dialectical Behaviour Therapy] but I wasn't properly engaging with it, because it's hard to understand what people are saying online whereas in-person, we'd have the worksheets in front of us and we'd have pens out… So, when we couldn't do that, it felt like a waste of time really… I feel like worksheets should be sent in the post. I remember she [referring to therapist] wanted me to print things out but I don't have a printer… I feel like I'm letting her down and I'm not doing good enough when I can't print something out (CYP 08).

Healthcare professionals and young people highlighted the differences between group sessions delivered in-person and online, with young people describing the latter as ‘boring’ due to a lack of interactive elements:

I used to run DBT groups, and a lot of things that would make it really engaging involved things like props that we could pass on. For example, one of the tasks we used was holding something from the freezer that’s really cold to touch… It’s really rubbish the way we have to translate that virtually, it’s just saying ‘can you grab the nearest object or something that you can hold’… I think it's good when it’s something really cold and switching it between people can be quite fun, whereas that was really lost in therapy online… I know DBT would work better in-person… It's a lot of tactile things and ways to make DBT fun. There’s no way you can do that online (HCP 03).

It was a bit boring being online and doing stuff online rather than face-to-face because then you are actually moving and it’s more physical (CYP 05).

Healthcare professionals also reported difficulties recreating group sessions in the online milieu. As some young people prefer to type their response, this presented additional challenges as clinicians were simultaneously expected to manage the chat function whilst delivering the content:

With that group work, especially on Teams, you're juggling two things. So, you're delivering the actual group, but then you've got to make sure you keep an eye on the chat as well… Talking at the same time and being mindful of the chat, I find that quite cognitively demanding (HCP 03).

They type rather than speak out loud if you ask a question. You then have to sit there awkwardly for a minute or two, and then you have to gauge is anyone actually typing a response… The group sessions don’t work at all (HCP 01).

Clinicians have also struggled to recreate the important social aspects associated with in-person provision, such as the provision of refreshments. For example, 'you will also lose the tea and coffee time' (HCP 02). This aspect of provision is important as one young person reported attending in-person group sessions before the global pandemic ‘for the food’ (CYP 05). Spontaneous peer-to-peer interactions cannot be satisfactorily supplanted online:

It's going to be difficult for them to engage next to each other and choose who they engage with and engage individually in a way that we would ordinarily, you know, when a couple might pair off and have a discussion about something they might have heard in the group wouldn't be able to do that online necessarily (HCP 02).

In addition to difficulties facilitating group sessions with children and young people, healthcare professionals also highlighted the challenges associated with delivering family therapy in the online milieu (‘If I’m seeing a family, you don't necessarily get all of the family on the screen. That can be a bit of a challenge’ (HCP 06)). According to young people, family therapy does not work well online (‘Family therapy was awful online’ (CYP 08)). During in-person provision, therapists can more clearly observe family dynamics:

If they go into a physical space, for any number of reasons, they might position themselves where they're not sitting next to each other… If they were in a physical space, you might actually comment on that… Whereas you may be less inclined to say that if they're scrunched up on a couch. What's lost as well is maybe movement, activity… So often, the young person or the family are just sat in front of the screen and I probably do the same whereas sometimes you might be more active [in-person] (HCP 06).

### Practical issues

Many participants reported interference due to connectivity issues. Unstable internet connections resulted in disrupted therapy sessions, which presented challenges for both young people and healthcare professionals alike. For young people, technological disruptions interrupted their flow, presenting an additional layer of difficulty:

It also felt a bit disconnected in a way because online internet fails, and you'd have to do just wait and then it's like you've gone off topic… If you’ve gone off topic then going back would be hard (CYP 06).

Many young people feel anxious before their therapy session, and experiencing technological disruptions or failures could further exacerbate such feelings:

If that happens to a client [referring to connection issues], it already puts them in like a really stressed state of mind when they're already doing something really challenging in seeking mental health support… Sometimes if you're on a Zoom call, and you can't find the password… it makes it really stressful for you and going to mental health support is already stressful enough (CYP 04).

If a child is anxious, the anxiety can build a little bit while they are waiting to have the meeting or if the meeting drops because of signal (Parent 01).

Healthcare professionals shared similar thoughts, referring to such issues as ‘time consuming’ (HCP 02) resulting in additional work. For example, ‘it freezes, it crashes, then the appointments gone and you're going to have to rearrange (HCP 04). Issues with technology also impact clinicians’ competence delivering virtual sessions:

Technical issues have been an issue… There were times when the sound doesn't quite work. There were times when you're not getting linked up properly or the young person's internet isn't working (HCP 02).

A lot of the time I’ve had tech issues. So, I do a lot of groups and a lot of young people tend to have trouble going on to links and that really puts me off my flow. I feel like I almost get a bit flustered (HCP 03).

### Losing the cues

Although ‘90% of our communication is non-verbal’ (HCP 04), healthcare professionals found it challenging to ‘pick up’ or identify non-verbal forms of communication during remote sessions:

A lot of what I do as a therapist is about nonverbal and non-spoken communication, so the nuances of a body language is lost. I think that applies maybe to whoever is receiving the therapy too. They might not necessarily pick up on some of my nonverbal cues, clues, communication (HCP 06).

I think we do miss things non-verbally. I only see your head and shoulder… I can't see if you're holding your stomach now, because you're really anxious, or you're fidgety in the seat, you know, those very subtle movements that you might have that might indicate you're in distress, you're upset, you're hiding something, that you as a therapist might pick up (HCP 04).

Young people highlighted that non-verbal communication is a powerful source of insight in therapy as clinicians can learn about their feelings through observing their body posture, eye contact, and gestures. Often non-verbal communication is incongruent with spoken words, and young people and parents/carers acknowledged that this is harder for clinicians to observe in the online milieu:

I don't like to talk a lot about my mental health but sometimes the clinician can see if I'm folding myself up or if I'm not maintaining eye contact, that maybe means I am anxious about a certain issue or there might be something that needs to be explored more whereas that's harder to do on a 2D surface on the screen. So that requires the person on the other end to open up more and be more literal about themselves, which is really challenging (CYP 04).

I have bad anxiety but if you look at my face, you'd think that I’m a really happy, bubbly person but below the camera, I'm fidgeting, and I can't stop my leg from bouncing… I feel like you can’t really be understood properly over the phone like you can in-person (CYP 01).

The therapist might have picked up on his cues a bit more easily if they had of been face-to-face… Quite often he'd be quite fidgety, and you can't really see that on a screen (Parent 02).

Children and young people also experienced difficulties when trying to read their therapist’s body language and facial expressions, and such difficulties impacted their feelings towards remote provision:

I hate it [remote provision] so much… You just can’t get the body language from people, especially if it's someone you've never met in real life. You can't get them the same as if you were to be in-person with them (CYP 07).

You can't tell their body language… It's more like you can't see what's going on, and you don't know how they're reacting to what's happening because really you just only see the face. It's harder for people to get through to other people as well on it. I think in-person appointments are so much better than telephone ones or Teams ones (CYP 01).

I think it was just harder work for him to do it online because you've got to read people's expressions through a screen, and you can see yourself which is weird. And there is sometimes a slight delay in talking and the other person hearing what you're saying, and you crossover (Parent 02).

Young people experienced similar difficulties when participating in group sessions remotely, with many highlighting their struggles with turn-taking online. For one young person, these difficulties led to non-engagement:

I don’t usually go to the group ones at the moment because I find them hard to do… I find it difficult to talk in a group on video because it’s harder to almost catch social cues or when the next person is going to speak or when to speak. Normal social stuff is made impossible by video calling (CYP 05).

As young people can see themselves on screen, they may alter their facial expressions and the interaction becomes much more managed than it would in the usual therapeutic environment, which adds an additional layer of complexity. One professional referred to this environment as ‘a more stage-managed setting’ (HCP 01) as often young people ‘present an image online’ (HCP 04). Clinicians are also more aware of their own body language and facial expressions when engaging with young people online:

Being more conscious of my position, if you like, in terms of my body language and how I might present. So, I'll look up at myself sometimes on the screen to check my emotional expression, for instance, or how I might be presenting (HCP 06).

However, many children and young people do not opt to turn their cameras on, or if they do, ‘you only see a little section of their face’ (HCP 09), which makes it impossible for clinicians to pick up on non-verbal cues:

Where we had to be working remotely and that being then the choice between telephone and video, I think lots of young people opted for telephone because they didn't want to see themselves on video… We're talking about often quite difficult things and thinking about when people are at their most vulnerable, and they might appear the most vulnerable and then that's very visible if they can see that and very aware of somebody else seeing that (HCP 09).

Although for some young people this is a choice, others do not have access to a computer or laptop with an inbuilt camera: ‘I know a lot of young people and families don’t have access to tablets and laptops and online’ (HCP 05).

Beyond the therapy room, healthcare professionals highlighted the importance of informal greetings and conversations that occurred in the waiting area and walking to the therapeutic space. Often young people have not yet ‘put their guard up’ (HCP 02) at this point, offering professionals an opportunity to pick up on subtle cues:

I think we lose the time when you're less focused on the assessment and the review. So, on the video link, you’re straight away face-to-face with the young person and starting a review. Whereas you lose that walking down the corridor bit where sometimes young people put on a face for a review and they might put their guard up, or they might prepare themselves in a certain way, and then you might find that a young person hasn't quite done that yet on the walk in or loses that face as they're walking away, and you can see them leaving in a slightly more relaxed way that you can then interpret (HCP 02).

When the sessions are ran virtually, it is the case of you're on a laptop, and you're just waiting for a young person to say login or appear or whatever, and then the session begins. Whereas the thing that I like in-person, which I think that has a big impact on the relationship as well, is it's not just the case of you know what therapy starts now and that's it. It's actually you're in the building, and there'll be sitting in their waiting room, and first you go up to them, and say hi, are you ready for your session? And then even just like little things, like actually walking to the therapy rooms (HCP 03).

As non-verbal communication is often relied upon to form clinical impressions and maintain engagement, healthcare professionals were concerned that subtle cues were being missed during remote sessions, leaving them ‘having to make lots of assumptions and inferences’ (HCP 09), especially during telephone sessions. As a consequence, healthcare professionals acknowledged that remote sessions were often shorter in duration, leaving many questioning their professionalism:

It's really difficult to get a sense of what's going on for the young people [remotely]. When I'm then seeing them face-to-face, I'm getting a very different perspective… I got to a point where they are just not working [referring to remote appointments], and I don't feel like I'm being safe practising remotely (HCP 07).

I feel like you’re looking at a more stage-managed setting with them because they are all sat very still. It’s difficult to pick up more subtle cues about them… I found that the appointments are faster when I'm doing it online, so I don't know if conversation flows less easily, or whether I don't pick up on the cues that they give me as much. But I definitely get through the appointments quicker. I’m worried that I'm perhaps having less detail when I’m making my decisions (HCP 01).

Thus, compromised professional competence was acknowledged. In line with this, some professionals reported finding it harder to manage risk and make decisions about risk when engaging with young people remotely:

I do sometimes feel more comfortable assessing and managing risk face-to-face than online… Last week, I met a young person for the first time after say three or four sessions online, and I met them face-to-face. We both said actually it feels better. That was a mutual agreement… From my perspective, it was around the management of risk. I don't know what it was, but when I met with her and there was some significant risk, it just felt that we connected more maybe… It was certainly something about risk for me and doing it online didn't feel as safe and I didn't feel as confident in assessing and managing risk than seeing them in-person (HCP 06).

Consequently, professionals acknowledged that they were ‘providing a lower quality service’. The training of healthcare professionals should stretch beyond the therapy room itself. Training staff to be attentive to non-verbal cues in online settings could enhance service provision:

I would need more training on the communication skills in an online environment. At medical school, we did loads and loads and loads of training about how to have your sessions, and how to pick up on all the cues that you would have when they're in the room with you and we don't really have anything like that for online stuff… So, I’m providing a lower quality service (HCP 01).

### Drivers of trust and rapport

Therapeutic relationships are one of the most important aspects of mental health care; however, difficulties forming relationships were acknowledged ('It is harder to form rapport with the client through a screen, and I think rapport building is one of the most important aspects of any kind of medical care' (CYP 04)). Young people were grateful to have established a therapeutic relationship with their clinician prior to the pivot to remote provision:

With online, you don't get anything out of it, you just sit there, and you don't really connect with the people… I feel like with online you don't get that connection. Fortunately for me, I already had my set people, but I can’t imagine just being referred to a system and not have met anyone and having to meet them for the first time over Teams or whatever. That would be hard because you can’t fully understand each other and you can’t connect (CYP 08).

Some clinicians found it hard to create the intensity of relationship required for trust and change without meeting in-person. As therapeutic relationships constitute the essence of successful therapy, many clinicians felt that face-to-face sessions were necessary to establish engagement and rapport:

It feels to me that you probably don't get as good a rapport. You don’t build up the same level of connection with them that you would do if you were doing it face-to-face. I speculate that they find face-to-face gives you a better relationship… The kids that I've seen face-to-face routinely, I've tended to have a better relationship with them than the ones I've seen only online (HCP 01).

Getting that engagement and rapport, I think, can be a bit more tricky [in the online milieu]… I just think my personal feeling as a professional, where our skills are not just in the words we say, but enormously in the human connection that we have with the young people, it's harder to get that quality of rapport (HCP 02).

Clinicians highlighted a sense of loss in respect of the personal connection and direct intimacy of meeting, which made silently providing comfort or support problematic. Being together physically and the tactile nature of in-person provision has a positive impact on the therapeutic relationship:

There's an awkwardness to video links… you can't touch them. So those that might need a hand on their knee, that can be really valuable and really powerful therapeutically when somebody that you've been talking to about something that means so much, and there's an element of touch that you won’t be able to get via a video link (HCP 02).

If you're really having a hard time, they can come close to you and tell you it's okay [when in-person]… When you cry, it just seems really awkward and weird via a screen (CYP 10).

Informal greetings and conversations on the way to the therapy room further support the establishment of trust, thereby having a positive impact on the therapeutic relationship. One healthcare professional suggested that informal conversations and showing a general interest in young people’s lives enabled their clients to feel comfortable disclosing and articulating complex emotions during therapeutic sessions:

One young person I worked with would have sessions virtually, but she wouldn't really share a lot and it was really hard to prompt her to share… When it happened in-person, even the first time that I met her in-person, it just was almost like a radical change, and this person did start talking a lot and I discovered a lot in a very short period of time (HCP 03).

In an attempt to establish therapeutic relationships remotely, clinicians afforded time during each session to talk about young people’s interests and hobbies. The appearance of pets on screen was also appreciated by young people, with some healthcare professionals suggesting that this helped to establish connections:

Online I was much more willing to be asking about the football and ‘oh you've got a dog’ to put the effort in. To put that extra 10 min of time or 15 min of time to get the relationship on track by using their interests (HCP 08).

I would tell them about my pets, and that was a really good way to avoid it becoming like stilted and stuff, because you have that shared thing to talk about and it helps you to connect as people (CYP 04).

Young people absolutely love it [when pets appear on screen]. I think because maybe they see that yes this person's a clinician, but they're also a pet lover as well like a lot of people are… I wonder if that's actually to do with the power dynamics as well, so they are not only seen as this clinician giving therapy or treatment, it's like, ‘oh that person has interesting pets’ (HCP 03).

Despite such efforts, new ways of working changed the experience of relationships and contacts for some young people. For example, one participant reported that the relationship with her therapist deteriorated following the pivot to remote working, which subsequently impacted negatively on the progression of her treatment:

I’d say definitely my psychologist, therapist, it definitely changed. I mean we weren’t really close but we had a decent therapeutic relationship but when it went online, I guess it went out of the window really. It was hard to get through to her and stuff. It did just change for the worse basically. Now I don’t see her at all really… So being online did really impact me and my DBT and my relationship with my therapist (CYP 08).

## Theme 2: Spaces and places of therapy

### Spaces of comfort and safety

As young people are familiar with technology and interacting with peers online, many participants suggested that the pivot to remote provision increased comfort levels, offering young people ‘a feeling of safety’ (Parent 01):

They're very much more familiar with the technology… and find it quite comfortable and easy to use (HCP 02).

As well as familiarity with the medium, interacting from within their own home environment also enhanced feelings of comfort and safety for some ('I feel like they felt quite comfortable in their own environment' (HCP 03)), which may have enabled young people to ‘open up more quickly’ (Parent 02):

I think it helps to not be in a clinic setting. To be in a familiar place you might feel more able to open up more quickly because you're not in a drab room (Parent 02).

I think for some young people, they're more likely to attend… They've not had to have the stress of walking into the building… So, I think people are more at ease, more relaxed in their own homes and they feel safe (HCP 08).

Young people also referred to their own space as ‘safe’ and a place of ‘comfort’ (‘I’m in my safe space all the time, which helped me’ (CYP 02)), which enabled them to feel more ‘relaxed’ and less ‘vulnerable’ whilst engaging in therapy remotely. It seems that engaging online can also afford young people a sense of control. For example, young people can control the pace of therapy through the initiation of breaks, such as switching off their camera or putting themselves on mute ‘to take a breather’ (CYP 10):

It's like a barrier online. It doesn't feel like you're exposing yourself, even though maybe you have somehow but it just doesn't feel like that. So, it's more safer… You’re in the comfort of your own home… Online, it was relaxed. I didn't have to pressure myself to act in a certain way. It literally felt like someone's on a screen let's just talk (CYP 06).

I also like online support, because when I'm seeing someone… I get super anxious saying stuff, you can always take your camera off to help your anxiety or if you want to take yourself on mute for a second to take a breather you can (CYP 10).

However, some young people felt less comfortable using this medium to converse ('I just don’t think she felt as comfortable as if it was a face-to-face' (Parent 01)), and others reported that they did not feel as though talking to someone via a screen was a ‘real therapy session’ as online sessions often felt ‘too relaxed’. Professionals also shared these views: ‘It doesn’t feel like a proper therapy session for us’ (HCP 08). Such feelings may have had a negative impact on treatment outcomes:

Whenever you're talking to someone in the present, not through the internet, it's like a vulnerability comes out…When it's online, it's like you don't have that on the spot vulnerability… I'd say it didn't really meet my needs, because sometimes it was like maybe this is a bit too relaxed (CYP 06).

I think face-to-face is more challenging. So, it’s potentially a bit more therapeutic to be face-to-face… The anxious groups that would tend to avoid interactions and therefore will be quite comfortable online, but actually need to be challenged and from a therapeutic perspective, need that exposure to the real world really in order to start to work on their difficulties (HCP 02).

### ‘When you’re at home it’s home time’

As young people were engaging in therapy from their home environment, there were numerous distractions, which often led to difficulties in concentration. For example, ‘when I’ve got people walking in or disturbing me, it’s really hard to concentrate’ (CYP 08). Conversely, when attending sessions in therapeutic spaces, young people’s attention is directed:

It was harder to focus in my home, because there’s a lot more distractions. A lot of therapy rooms at [CAMHS building name]… have just a white wall and a table and a chair… Whereas at home, there's like way more stuff, and it's harder to focus in an environment that you've been conditioned to do one thing in. It would be like if I did my washing up at the cinema where I work (CYP 04).

You can’t engage. Online is just hard. It’s just not a proper therapy session, because you're just stuck at home. I think there's something about going into a place and it feels professional, and it feels like you're actually going to get something out of it. Whereas being at home, it just feels like ‘oh it's just a meeting and it'll be over soon’. You don't have to put as much effort into it, so your brain is not as simulated and ready to concentrate… It feels like when you're at home it's home time. But whereas when you go out, it's like it's therapy time. Your brain knows that (CYP 08).

As online sessions were not perceived to be ‘real therapy sessions’, young people did not afford the same level of effort. Conversely, the therapeutic environment is professionalised with a code of practice that is sacred to it. In-person therapy requires more ‘effort’ as young people are required to get dressed and travel. As young people must work towards functioning well in the outside world, these aspects form an important part of the process:

When people are coming to CAMHS, they know they are coming to a quiet safe space, and they are guaranteed to get that, and they are in that frame of mind. Whereas sometimes they have just turned off the TV and turned on their phone to do a video call… Online is sometimes a bit too easy. If you say you’re going somewhere, right away you would put a bit more of an effort in because you’ve got up, you’ve got dressed and you know the purpose that you’re coming for… Maybe the focus and concentration isn’t there [online]… I’ve had many sessions with kids, and they are looking down and they’re on their phone you can tell that they are texting, and they are snapchatting other people and they are just not fully engaged (HCP 05).

It seems common for young people to use their smartphone when attending remote sessions, which can result in distractions and disruptions, such as phone calls or notifications:

Often they use their phone and then their phone might ring and then it’s like what do they want and then their mind is like what are they calling me for or if they’re anxious they are then worrying or are embarrassed (HCP 05).

When accessing it on a mobile phone, sometimes you get text messages come up and your camera will go off and your clinician will be like ‘oh where have you gone?’ But it's just because you've got a text message, and you can't really help it. Even if it's just a notification pops up your camera turns off… During the first lockdown, I had all of my therapy on my phone. That was challenging (CYP 04).

Young people also reported that clinicians seemed distracted when delivering therapy from their home environments. These thoughts were mirrored by healthcare professionals who also reported concentration difficulties when engaging remotely:

You're in own home. For example, I have got a little baby, and she's not in nursery on a Monday or a Wednesday. So, I can hear her sometimes crying in the background and I can hear her when I’m on call, even though they can’t hear her, I’m put off because I’m trying to concentrate but my daughter is upset (HCP 05).

They were getting distracted by the dog and then someone would come in the room. And then I just feel like they weren’t fully focused, and they were giving a different kind of therapy because they couldn’t say too much either because there were people in their house (CYP 07).

I also found that the quality of the therapy overall on online / remote therapy, I don't know if it's just me, but it seems to be generally a little bit lower as if the therapist doesn't really seem to be fully engaged with it as much as it would be, and to be fair, I feel like that as well. I feel like I can't really engage properly remotely and online. I feel like there's just something lacking (CYP 09).

Last, healthcare professionals raised concerns for both themselves and their clients following the conclusion of remote sessions, as there was no physical separation or transition between a therapy session and ordinary life. As difficult feelings ‘bleed out’ into the wider environment during therapy, issues arose for some participants in terms of moving on from a session after it had concluded:

If we think about therapy, the difficulties might be at home… So if you do therapy and you go to a clinic and you bring some stuff up, you can walk out and leave that behind. Whereas if you’re in your own personal space or your home, you do therapy, and it stays in that room [or house]… When we are in a clinic, we can do a session, and then we might be able to speak with another colleague… But if you're in your home, it almost feels sometimes that you can’t separate… Working from home/ doing stuff online can have its own challenges in terms of detaching (HCP 06).

It’s nice to just have those corridor conversations or to have a cup of tea with someone or to go for lunch with someone. Because of the nature of the work, you’re working with people who want to kill themselves, so you just need to take 5 / 10 min to yourself but if you’re at home it’s not the best. So that’s certainly one of the challenges, managing your own wellbeing (HCP 05).

This is especially important when considering that some young people referred to CAMHS have experienced trauma:

Personally, when they were reliving, remembering trauma and they were in another part of the city, it just didn't feel as held… There was one young woman that I worked with who had asked specifically for EMDR [Eye Movement Desensitisation and Reprocessing]. She didn't have the best support network round her. That left me thinking should I have done this?… I think with that level of trauma again, with a young person, I probably wouldn't do the EMDR online, not with that level of trauma (HCP 08).

### ‘You lack privacy to speak about things’

Engaging in therapy requires a safe and private space where a young person feels able to speak freely. In relation to remote provision, healthcare professionals acknowledged privacy and confidentiality concerns, as some young people may not have a private space in their home environment. Conversely, when attending appointments in CAMHS buildings, young people are ‘guaranteed that private space’ (HCP 05):

I was trying to do an appointment the other day [online], and somebody kept walking into the room. At one point the person walked into the room just as I was asking about suicide and risk. How can you be honest and open in that situation? … There's something probably about sitting there and trying to talk about things that are quite difficult for you in a space where you don't necessarily have the privacy. Here [referring to CAMHS building] I can guarantee that. I can ask the parents to leave, and the parents aren't going to overhear what's being said (HCP 07).

Young people also highlighted privacy concerns when engaging in therapy from their home environment, which impacted upon their willingness to ‘open up’ about difficult life experiences and/or articulate complex emotions:

I feel like it's not confidential… What if my sister is outside the door or what if they can hear me downstairs? It stops me from opening up as much as I would want to (CYP 08).

When you do it at home, especially if you've got family in the house, you might not want to talk about something if your family are in the room (CYP 07).

Thus, engaging in a therapy session at home does not afford young people the same level of privacy and confidentiality as engaging in a clinic session as ‘sound travels in a house’ (Parent 01), which can result in parents or carers unintentionally overhearing information shared:

Sometimes they may want to speak about things that they don't want other people to hear, and being in your home, it's easy for people to overhear what you’re talking about… When [she] used to go for meetings with [her therapist in-person], she would go into the room with [therapist] and I’d sit out in reception, or I’d pop out for half an hour… So, there was total privacy for her to speak how she wanted to and to say whatever she wanted to say. Whereas I think when you’re in your own home, I could hear some of the meetings that she was having (Parent 01).

This is especially important when considering that it is common for young people to be referred to CAMHS following adverse childhood experiences involving their parents or guardians. Talking through these difficulties in the environment where the adversity may have occurred could present difficulties for the young person, especially as parents could overhear:

I always think about what the parents might think about their young person having a session in their room… They're going to know that their child will be talking about stuff that their parent did and lots of conflict. What's really common in CAMHS is a lot of sexual abuse… The parents will know that the young person is probably going to say, ‘well I experienced this, because my mum was in a relationship with this person’. So, a young person is doing their session and the mum could be in the home tidying up (HCP 03).

## Theme 3: Future of CAMHS

### Moving toward a hybrid model of provision

Some young people prefer face-to-face provision, whilst others prefer the digital alternative; a ‘one size fits all’ strategy may not be appropriate following the pandemic:

She really doesn’t do well with Zoom or phone calls. She tends to just answer the questions they are asking her. She won’t interact. I think the face-to-face works so much better for her (Parent 03).

When I asked him about his experience, he said, ‘I would probably have never engaged with you if you'd asked me to come to the clinic’… We have to think about the individual needs/preference… Doing it over the telephone and then graduating to online, but the camera off really worked for him (HCP 06).

Engaging in therapy via remote means does not work for all young people, especially those who are younger and/or have neurodevelopmental conditions:

We do work with the children, and I’d say up to the age of 13 I don’t think a lot of those young people have those skills over the phone or video. I don’t think it’s something children can easily do… The younger people struggle, particularly people with neurodevelopmental conditions such as ASD [Autism Spectrum Disorder] and ADHD [Attention Deficit Hyperactivity Disorder] (HCP 05).

So young people who have any neurodevelopmental conditions, say autism for example, or any sort of learning difficulty, I think they tend to struggle with virtual working (HCP 03).

Nevertheless, alternative modes of provision are advantageous additions to service as usual. The complexity of the balanced preferences shows how a hybrid system based on individual needs should be the outcome. Young people, parents/carers, and healthcare professionals believe that services should plan for a rebalanced provision with greater online capacity, with CAMHS implementing a ‘laptop loan scheme’ for children and young people who would like to access therapy remotely:

I would take a blended approach unless somebody expressed a clear preference for one, and then they could be given that. But in some cases, like some clients, they might prefer online. But you might notice that they have a communication difficulty, or they might learn better in-person. And you might have to do all in-person sessions. If you're going to require people to use online services, I think you could have like a laptop loan scheme or a mobile loan scheme, just so that people can access that (CYP 04).

I think a blended approach would be a good idea. It depends on the child and the young person as well… I think it would be a good idea to ask the young person and the family, ‘what approach would you like?’ (Parent 02).

I personally like being face-to-face but other people might like being at home… If I was to look at CAMHS, you want the option. You want some people to be able to do online if they want to and people to be able to have face-to-face when they want to (CYP 08).

### Efficacy

Perceived efficacy is context dependent. With regard to individual therapy sessions, healthcare professionals suggested that children and young people should be given the option of accessing support either in-person or remotely:

There will be an element of patient choice and whether they want it in that style, but then there will be times when a clinician may be clear that they can't really do the therapeutic work effectively without the face-to-face (HCP 02).

Obviously face-to-face you get a bigger, better, more accurate picture, but I know there is research that suggests that interventions that have done CBT just with people over video, and it has been proved to be effective. So, it does work for some people, but certainly not for all… I found that it is effective [referring to online CBT]. I wouldn’t say it’s better. I think it depends on the individual. I’d say under 12 it’s best face-to-face. Thirteen plus I think video calls are probably just as effective. Not better (HCP 05).

Family therapy does not lend itself to virtual therapy as professionals found it harder to include everyone in the process. Similarly, group sessions were perceived to be more effective in-person. When restrictions allow, it will be beneficial for group sessions and family therapy to resume in-person:

With regard to family therapy, I do think that in-person remains still the preferred modality or the preferred way of working. I think it has to be about choice with the family. But I think we'd also have to think as a team about whether it was useful or not, or effective as it could be. So again, we could still give families choice. But we would have to make a clinical decision about whether we thought there was a difference between how things were online, and how they could be in person. So it's a fine balance really about providing choice, but also thinking about clinical effectiveness. Moving forward, I would say that we would probably be inviting our families to or even encouraging families to attend in-person. But if there was no option for that, we certainly wouldn't exclude a family from family therapy (HCP 06).

Group working and virtual working, I feel like it’s a big no no. It's hard. Young people and virtual work, I just don’t feel like you could just get the most out of anything (HCP 03).

Many healthcare professionals acknowledged that face-to-face provision is superior (‘I just think we're not being as effective as we could be if we were seeing someone face-to-face’ (HCP 06)), with some even suggesting that all provision should return to face-to-face due to the myriad of benefits associated with being physically present together in a therapeutic space:

It [online therapy] will never be as good as face-to-face… Clinically deep-down face-to-face probably has the edge (HCP 08).

I don't like online working. I think when I have tried it, more often than not, it's not worked. I think sometimes it's probably reduced the demand, because kids aren't engaging. So, then they are being discharged… Face-to-face should be our forward-facing approach, but the option for remote should be there and actually, it should be down to the person's preference (HCP 07).

I think the default position has to be everything is in-person, but if the young person does request that they might want virtual sessions, then yes, we can accommodate it… The main reason for this is that it [online provision] gives young people too much of a space to hide (HCP 03).

While evidence suggests that some forms of therapy appear to work effectively online, one healthcare professional felt that effectiveness of the delivery processes for the therapist as well as the relative benefits to service users of each mode of delivery ought to be explored before any decisions on modality were finalised:

I suppose we do need… to evaluate how effective it is. What's effective? Who is it effective for? Why is it effective? (HCP 06).

### Increased demand: is social prescribing a solution?

All CAMHS services are experiencing increased demand, resulting in young people facing longer waiting times and others being discharged after only a limited number of sessions:

Unless they are seriously trying to take their own life then they are not seen as a risk… I wouldn’t discharge them as quick as [name of CYP] has been discharged. She hits herself… and she digs her nails right into her fingers where they bleed. I wouldn’t class that as minor… I just think the service are on a tight budget and they’ve got to prioritise the kids who are in serious need of doing real self-harm to themselves. I think each child should be given more time (Parent 03).

The length of waiting times has gone up drastically. So, the number of people seeking CAMHS help has increased… I think there's probably an element of, for some kids, school was the problem and for other kids, home is the problem and for other kids, it's just being in a pandemic. All of that is combined into one perfect storm so demand seems to have gone up quite considerably for I think all of the young people’s mental health services (HCP 07).

As all CAMHS services are experiencing increased demand following the COVID-19 pandemic, one healthcare professional emphasised that a ‘sea change’ (HCP 08) is urgently required:

I think the kids who are accessing the mental health services now because of COVID, it’s scored through the roof (Parent 03).

There was a leaflet in the GP surgery, and it said social prescribing and I just thought ‘that’s it’. Our waiting lists are months long, and children are waiting in distress for help. So, I thought here is a way of helping children to have more child friendly interventions and to get seen more quickly (HCP 08).

In light of this, CAMHS have innovated and are in the process of developing a social prescribing offer for children and young people:

We are developing a social prescribing offer which includes horticultural therapy, forest schools, arts, drama, physical activity for all of those kids who can’t sit still and just talk, of which there are vast quantities. Actually, talking and doing something at the same time, particularly for boys is probably better (HCP 04).

We've got this massive project about to launch… with Forest Schools, horticultural therapies, and this thing called Nature well. So, it's [referring to COVID-19] enabled us to think outside the box massively… There's going to be a massive sea change here because there is stuff about credibility [as] parents want a young person to see the psychiatrist or the doctor and we're saying ‘no, let's get them out in the sticks’ (HCP 08).

As many young people who access CAMHS are socially anxious, this project will provide access to opportunities that may not otherwise have been accessible to them. Following completion of courses, young people will be encouraged to access opportunities in their local community:

The social prescribing is art and outdoors… And then there's the community part of it, which is the proper social prescribing, so a lot of our kids won't rock up at some community thing, they won't go to the community thing because their anxiety is so great so that’s why we have created basecamp. The basecamp is doing these outdoor and arts-based things, with us basically but then hopefully we pass them over to the community-based services at some point (HCP 08).

Creative and outdoor pursuits facilitate feelings of accomplishment and achievement as young people can develop their artistic abilities and work towards a goal in the form of a Duke of Edinburgh award:

A lot of the kids that we work with, if they're on the autistic spectrum, they often love art, that is the thing that calms them… The other thing we might do is I’ve had a long conversation with the Duke of Edinburgh award in Liverpool, and we think we're going to get a licence with them so that whatever the children are doing with us, they'll be getting some brownie points as well. There'll be getting a bronze or something like that. It's motivating, isn't it? (HCP 08).

## Discussion

This study set out to understand young people’s, parents/carers’, and professionals’ experiences of remote provision across CAMHS in one NHS trust in the North West of England to inform subsequent service delivery. Our key findings fall into two main categories: remote therapeutic experiences and appropriate spaces for therapy.

CAMHS have innovated rapidly, notably with accelerated digitalisation of services in their array of forms and formats. Remote delivery involved assessments and therapeutic intervention sessions via telephone or video call. Participants reported that having the flexibility to use different mediums improved access. Appointments were no longer limited by room availability at the hospital site or parent/carer availability, and attending appointments from home reduced travel burden and the associated costs (e.g., public transport fares or petrol). Although remote provision increased flexibility within the service, the therapeutic experience was altered [14]. Non-verbal cues are instrumental in assessing and engaging young people who are experiencing mental health difficulties. In line with previous research (e.g., [15]), clinical staff reported difficulties identifying non-verbal cues remotely. Consequently, appointments were often shorter, leaving some professionals questioning their professionalism and feeling as though they were providing a lower quality service. While previous research suggests that fruitful alliances can be developed online (e.g., [11]), remote provision adversely affected the therapeutic relationship for some young people in the current study. In line with previous research (e.g., [15]), many professionals felt that face-to-face appointments were necessary to establish engagement and rapport, especially as healthcare professionals reported spending more time building rapport when working online. This finding aligns with previous research (e.g., [10]) suggesting that the therapeutic process differs online, as healthcare professionals often spend more time building rapport than accomplishing tasks. In light of this, face-to-face remained the preferred type of appointment for young people, parents/carers, and healthcare professionals. Of the two virtual modalities, video sessions were preferred, with young people and professionals reporting that telephone sessions were least effective.

Although some young people suggested that remote provision increased comfort levels, others felt their place of comfort and safety was invaded. As there is no physical separation or transition between a therapy session and ordinary life, young people were left with strong feelings to manage alone in their home environment. When attending sessions in therapeutic spaces, young people are required to travel, which provides thinking space and an opportunity to pause in-between activities. Although attending in-person provision requires more effort on behalf of the young person, this is an important part of therapy insofar as it affords young people a sense of working towards a goal. Conversely, remote sessions were not perceived to be ‘real therapy sessions’. Further to this, context prompts the type of attention that young people naturally devote to a session. When engaging with their clinician in therapeutic spaces, young people’s attention is focused, selective, and directed; however, when in the comfort of their own homes, young people’s attention appears distributed, with professionals appearing to observe a softer form of attention. All participants reported connectivity issues, which had a negative impact on engagement during sessions. It is also common for young people to use their phone to engage in therapy, which often results in disruptions due to phone calls and notifications. Following adverse childhood experiences involving parents or carers experienced in the context of the home, young people may perceive their home environment as a place of trauma, which may impact treatment in unpredictable ways. It is also important to consider privacy and confidentiality as often parents or siblings are able to overhear.

### Implications

These findings have implications for mental health services worldwide where the option of remote provision exists. With regard to mode of delivery, our findings suggest that a ‘one size fits all’ strategy may not be appropriate following the pandemic as some young people prefer online provision, whilst others were keen to re-engage in-person. Although many healthcare professionals acknowledged that face-to-face provision is superior, especially in relation to establishing and maintaining a positive therapeutic alliance, remote provision may nevertheless be a better option for certain groups of CYP, according to their individual circumstances. In light of this, digital provision is no longer considered to be a peripheral way of accessing support. Healthcare providers are planning for a rebalanced provision, with remote provision becoming part of a menu of choices for young people and families. Enabling choice must be carefully considered on an individual basis, especially as in-person provision may be more therapeutic for certain young people. However, as remote sessions are only accessible to young people and families who have the relevant hardware, there is a risk of services becoming less inclusive. In considering future models of provision, the preferences, needs and capacity of the CYP, the accessibility to the parent/carer or family, and the professionalism of the therapist to identify the most appropriate format in each specific context are all critical when deciding which modality to use for the best outcomes for each individual. Thus, decision-making, on a case-by-case basis, should consider the wider context for each individual, as there will be circumstances that preclude face-to-face provision for some CYP (e.g., inability to reliably travel to attend sessions for financial or other reasons, diagnosis-related matters etc.). Although it is important to consider and understand the reasons underpinning each service user’s preference, a final decision regarding the appropriate format should be reached following a discussion between the healthcare professional and the CYP (alongside their parent/carer if/when appropriate). If services are to continue operating remotely, a ‘laptop loan scheme’ should be implemented, as some young people and families do not own relevant hardware. Young people valued the interactive nature of face-to-face sessions, whilst the online counterpart paled in comparison. Where possible, efforts should be made to make online therapy sessions interactive. Sensory resources and worksheets should be sent via post so that children and young people can use these resources during virtual sessions. In addition to this, non-verbal cues are instrumental in assessing and engaging people who are experiencing difficulties; thus, the training of mental health professionals should stretch beyond the therapy room itself as training staff to be attentive to non-verbal cues in the online milieu could enhance service provision [17]. Finally, perceived efficacy is context dependent. If services are to continue offering remote provision, trials exploring clinical effectiveness should be prioritised.

### Limitations

First, as data were collected from one NHS trust situated in a small geographic region of England, the findings may not be generalisable to other areas, particularly remote rural areas where broadband may not be available. Second, as all interviews and focus groups took place through virtual platforms, our sample may represent those who are more likely to be engaging well with virtual treatments. Nevertheless, mixed views on remote provision were elicited. Third, as with any qualitative study, the findings of this work reflect a small, fairly homogeneous group of individuals. Noteworthy here is that the CYP in this sample were predominantly female. In addition to this, although CAMHS supports children as young as five years, all of our participants were aged 15 years or older. Future research should therefore endeavour to incorporate the voices of younger children and young men. Similarly, as only three parents/carers took part in this study, future research should explore parents/carers views on remote provision.

## Conclusions

Although remote appointments increased flexibility within the service, the quality of the relational experience was altered. Reduced travel time for both clinicians and families may increase capacity, enabling the service to meet the increased demand if clinical effectiveness can be preserved. It is important to identify professionals’ training needs and determine how to support children and young people who experience problems with access or engagement. In considering future models of provision, assessing clinical need, patient and family preference, and access to space and hardware are all critical when deciding which modality to use for the best outcomes for each individual.

## Data Availability

Qualitative data extracts are presented in the article to support the findings. The data generated and analysed during the current study are not publicly available as the data collected is sensitive and could compromise the confidentiality and anonymity of the participants but are available (limited) from the corresponding author on reasonable request.
